# A new method to reconstruct the direction of parent-offspring duo relationships using SNP array data and its demonstration on ancient and modern cultivars in the outcrossing species *Malus* × *domestica*

**DOI:** 10.1093/hr/uhab069

**Published:** 2022-01-19

**Authors:** Nicholas P Howard, Eric van de Weg, James J Luby

**Affiliations:** 1 Institut für Biologie und Umweltwissenschaften, Carl von Ossietzky University, Oldenburg, 26129 Germany; 2 Department of Horticultural Science, University of Minnesota, St. Paul, 55108 United States of America; 3 Plant Breeding, Wageningen University and Research, Wageningen, 6708 PB The Netherlands

## Abstract

Unordered parent-offspring (PO) relationships are an outstanding issue in pedigree reconstruction studies. Resolution of the order of these relationships would expand the results, conclusions, and usefulness of such studies; however, no such PO order resolution (POR) tests currently exist. This study describes two such tests, demonstrated using SNP array data in the outcrossing species apple (*Malus* × *domestica*) on a PO relationship of known order (‘Keepsake’ as a parent of ‘Honeycrisp’) and two PO relationships previously ordered only via provenance information. The first test, POR-1, tests whether some of the extended haplotypes deduced from homozygous SNP calls from one individual in an unordered PO duo are composed of recombinant haplotypes from accurately phased SNP genotypes from the second individual. If so, the first individual would be the offspring of the second individual, otherwise the opposite relationship would be present. The second test, POR-2, does not require phased SNP genotypes and uses similar logic as the POR-1 test, albeit in a different approach. The POR-1 and POR-2 tests determined the correct relationship between ‘Keepsake’ and ‘Honeycrisp’. The POR-2 test confirmed ‘Reinette Franche’ as a parent of ‘Nonpareil’ and ‘Brabant Bellefleur’ as a parent of ‘Court Pendu Plat’. The latter finding conflicted with the recorded provenance information, demonstrating the need for these tests. The successful demonstration of these tests suggests they can add insights to future pedigree reconstruction studies, though caveats, like extreme inbreeding or selfing, would need to be considered where relevant.

## Introduction

Pedigree reconstruction studies using genetic markers have been conducted for several asexually propagated crop species and have elucidated many previously unknown relationships (e.g. [1–9]). The results of these studies have been useful for the development of new cultivars, genetic studies, historical research, and in the management of genebank collections. However, the seniority in some parent-offspring (PO) duo relationships identified in these studies could not be established. For instance, in a study to identify pedigree relationships between a large set of apple cultivars [7] using the Affymetrix 480 K SNP array [10], 407 PO duo relationships were identified, but 330 of them could not be ordered via the identification of one or both parents of one of the individuals in the duo relationship. The order was proposed for 202 of these cases when historical documentation indicated when “one member was clearly more recent than the other”. However, historical records are not always accurate or unambiguous, which could have resulted in incorrectly ordered PO relationships. Additionally, no order was proposed for the remaining 128 cases. In an extended diversity study of cultivated grape (*Vitis vinifera*) utilizing the Vitis18kSNP array, 490 PO relationships were identified, but attempts were not made to order them [6]. A similar study in pear (*Pyrus* spp.) has also identified PO relationships of unknown order [8].

Pairs of individuals are assigned a PO relationship when they have a common allele for each studied marker [7, 11]. Thus, barring caveats such as aneuploidy, parents and their offspring will share extended haplotypes across the entirety of their genomes. In diploids, the homologs that an offspring inherited from a parent will contain some level of recombination from the haplotypes from that parent [12]. These principles of inheritance have been successfully used in the validation and reconstruction of complex pedigrees where genotypic data is lacking for one or more of the ancestors [3, 4] and, similarly, were expected to be of use in the resolution of the order of PO relationships.

The ability to resolve unordered PO duo relationships would expand the results, conclusions, and usefulness of such pedigree reconstruction studies, including that of an ongoing large-scale pedigree reconstruction project in apple (*Malus* × *domestica*) [13] that was the impetus for this study. Hence, the purpose of this study was to develop and demonstrate methodologies to resolve the direction of unordered PO duo relationships by elaborating on the above-described general principles of inheritance patterns and recombination events.

## Results

### The parent-offspring order resolution (POR) tests

Two new POR tests were developed. They both require the presence of additional offspring for at least one of the individuals of an unordered pair. These relationships are needed to recognize and localize recombination events, which are what enable the tests. A highly curated SNP dataset and dense and accurate genetic map representing much of the genome are necessary for observing the recombination events for the tests. For the demonstrations of the tests, our dataset including 10 293 SNPs and the genetic map used were deemed sufficient because they met these criteria. The two tests are further elaborated on below.

### Parent-offspring order resolution test 1 description

**Figure 1:**
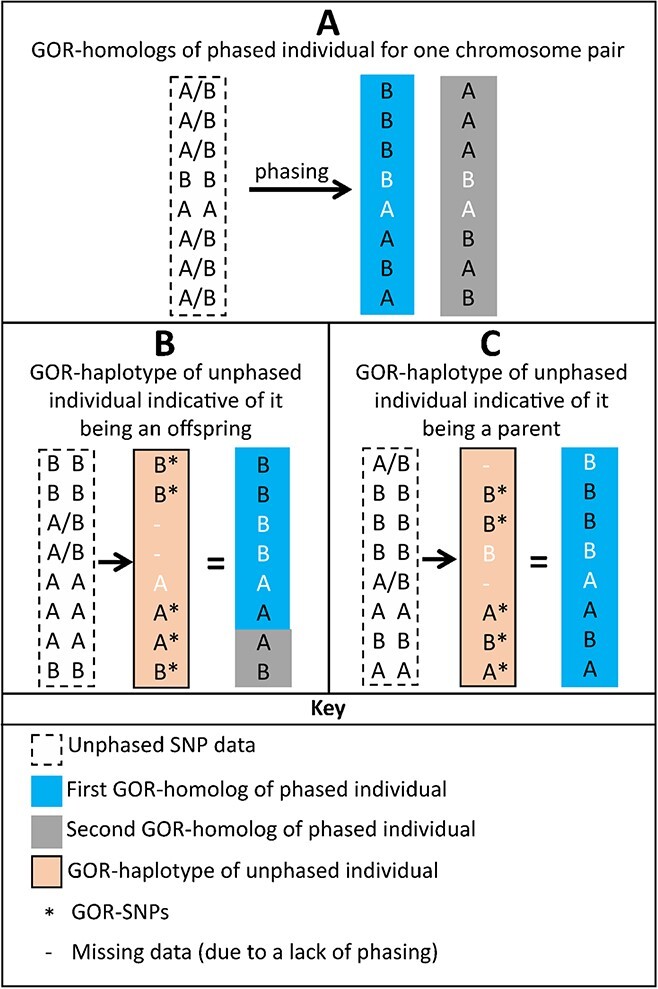
Schematic diagram of the POR-1 test. SNPs that are uninformative for the test are shown in white. Box A represents Generation Order Resolving (GOR)-homologs for the phased individual. Box B represents the GOR-haplotype of an unphased individual that is indicative of it being an offspring of the phased individual. Here the GOR-haplotype is composed of successive recombined fragments of the GOR-homologs. The unphased individual is an offspring of the phased individual when some or all of its GOR-haplotypes show such evidence of recombination of GOR-homologs from the phased individual. Box C represents the GOR-haplotype of an unphased individual that is indicative of it being a parent of the phased individual. The unphased individual is the parent of the phased individual if the GOR-haplotypes of the unphased individual consist of one unbroken GOR-homolog of the phased individual for each chromosome.

The POR-1 test requires accurate phasing data for one individual and unphased information from the other individual in an unordered PO duo relationship. The test is set up to probe whether some of the homologs of the “unphased individual” are composed of recombinant haplotypes from the “phased individual”. If so, the unphased individual would be the offspring of the phased individual, otherwise the opposite relationship would be present. This determination is made using what is termed in this study as “Generation Order Resolving” (GOR) information. There are three components of this information: 1) SNPs that are heterozygous in the phased individual and homozygous in the unphased individual, hereby termed “GOR-SNPs”, 2) the associated phased haplotypes for each chromosome pair from the phased individual, termed “GOR-homologs”, and 3) the haplotype from the homozygous SNPs from a chromosome of the unphased individual, termed a “GOR-haplotype”. The heterozygous SNPs in the phased individual distinguish the two chromosome-wide haplotypes of the GOR-homologs, while the homozygous SNPs of the unphased individual give unequivocal information on its GOR-haplotype. After defining the GOR-homologs and GOR-haplotype (as in Figure 1), the two are compared over each chromosome and the test is interpreted as follows.

The phased individual would be a parent of the unphased individual if a portion, roughly half, of the GOR-haplotypes of the unphased individual would be composed of recombinant GOR-homologs from the phased individual (Figure 1B). This is because in apple, one may expect slightly more than one cross-over per chromosome during meiosis (given the linkage groups are longer than 50 cM) and, as a recombination event affects only two of the four chromatids per chromosome pair, an average of half of the chromosomes in a gamete would inherit the recombination. An exception to this would be if one of the two individuals were fully homozygous for a chromosome, in which case that chromosome would be non-informative. Additionally, if there are no GOR-SNPs available on both sides of a recombination event in portions covering the ends of chromosomes, true recombination may not be observed, reducing the number of observed recombinant GOR-haplotypes. The phased individual would instead be the offspring of the unphased individual if one of the two GOR-homologs of the phased individual would fully match with the GOR-haplotypes for each chromosome of the unphased individual (Figure 1C).

### Parent-offspring order resolution test 2 description

**Figure 2:**
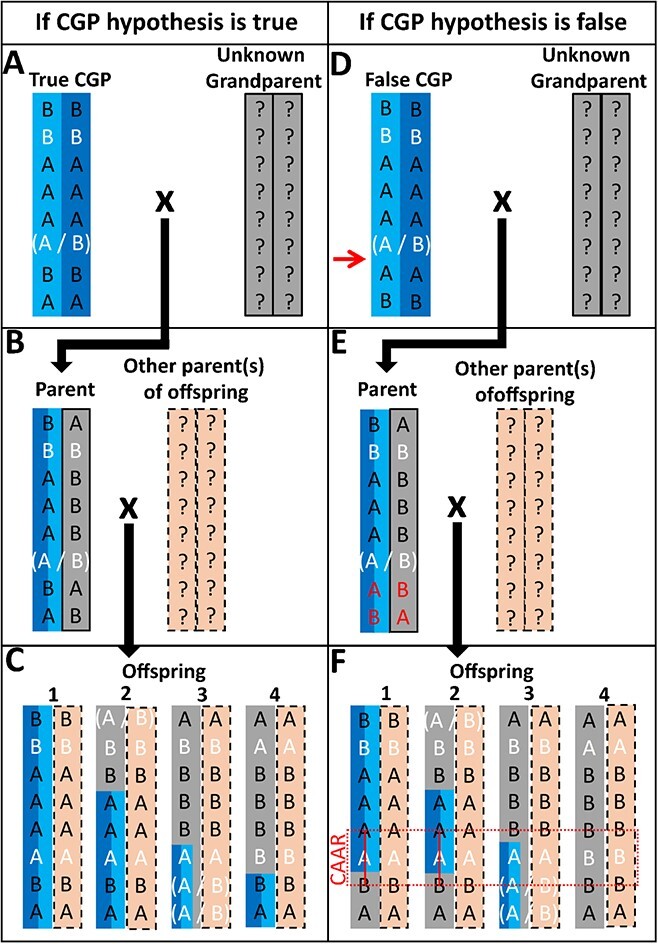
Schematic diagram of the POR-2 test. Boxes A, B, and C represent the test when the Candidate Grandparent (CGP) hypothesis is true and boxes D, E, and F when the CGP hypothesis is false. Each row represents a different generation that is used for the test. Allele calls in black are informative for the test, being homozygous in both CGP and at least one offspring and heterozygous in the Parent. Allele calls in white do not meet these criteria and are ignored. Heterozygous allele calls bordered by brackets represent uninformative, unphased SNP genotypes. The red arrow in box D represents the area in which recombinant parental gametes occurred in the CGP. The red alleles in box E represent SNPs with incorrect phasing deduction due to the CGP actually being an offspring and having received a recombinant gamete from the Parent. The red lines in box F thus highlight the common area of apparent recombination (CAAR) observed in offspring 1 and 2. The CAAR interval is between the 5^th^ and 7^th^ SNPs and is indicated by the dashed red line. Offspring 3 represents an example where the CAAR will not be noticed due to the lack of informative SNPs on one side of the CAAR (the lower part of the chromosome). Finally, offspring 4 represents an example where an offspring and the CGP both have a recombination event within the CAAR interval, masking the CAAR. These coinciding recombinations would only be confirmed in this instance via phasing of the SNP data coupled with the interpretation that the CGP is false.

The POR-2 test was developed for when insufficient phasing data was available for both individuals in an unordered PO duo relationship, but where at least two other PO relationships were available for at least one individual in the unordered duo. These relationships can be assumed as offspring in the test without negatively impacting the test results, but, if unordered, they could also be tested as possible parents through the test. The POR-2 test is set up with the hypothesis that one individual in a PO duo is a grandparent of the other individual’s progeny. The test compares expected and observed inheritance patterns along the three hypothetical generations: presumed offspring, the parent of those offspring (Parent), and the candidate grandparent (CGP). The Parent and CGP are the individuals in the unordered PO duo. To examine the inheritance patterns in a simple but efficient manner, the POR-2 test is based on a complexity reduction. The test uses SNPs that are heterozygous in the Parent and homozygous in the CGP and at least one of the offspring. This is the same concept as the GOR-SNPs from the POR-1 test. The heterozygosity of the Parent ensures that each GOR-SNP provides segregation information in the offspring. The homozygosity of the GOR-SNPs in the other two generations ensures that the origin and destination of each of the Parent’s alleles can be traced unambiguously (Figure 2), which makes that the recombination events that occurred in the formation of the offspring can be traced. For example, if the Parent was genotyped as *AB* and the CGP and an offspring as *AA*, the Parent’s *A* allele must have come from the CGP and been passed to the offspring. If instead the offspring would have been *BB*, the offspring would not have inherited any allele from the CGP but instead from the unknown second parent of the Parent. Recombinations become visible in the offspring through a switch in the grandparental origin of alleles of successive SNPs.

If the CGP hypothesis is true, the points of recombination in the Parental gametes that were inherited by offspring are the points at which the CGP haplotypes begin or end in the offspring. Their location on the chromosome will essentially be at random. In Figure 2C, offspring 1 has no recombination of parental gametes, which will be true in roughly one half of the chromosomes, as mentioned in the description of the POR-1 test. Offspring 2 Figure 2C shows recombination between the third and fourth SNP. The available data for offspring 3 (Figure 2C) do not allow for conclusions on the presence or absence of recombination due to the lack of informative SNPs at the lower part of the chromosome. The fourth offspring shows recombination occurring between the fifth and seventh SNPs; the sixth SNP is not used in the test because it is heterozygous in the CGP and thus uninformative (Figure 2C).

If the CGP hypothesis is false and the individual is instead an offspring of the Parent, a portion of the chromosomes of the CGP will be composed of recombinant haplotypes from the Parent’s homologs. This is depicted in Figures 2D, 2E, and 2F. The point where the false CGP inherited a recombination from the Parent is shown with a red arrow (Figure 2D). Each such true recombination would result in the incorrect phasing of each of the Parent’s homologs (indicated by the red allele calls in Figure 2E), which in turn results in false recombination in the offspring. This can be deduced by the observation of coinciding recombinations in the haplotypes of the offspring (indicated by the red line in Figure 2F). We termed these coinciding recombination positions common areas of apparent recombination (CAAR). A CAAR is apparent between the fifth and seventh SNPs in offspring 1 and 2 in Figure 2F. However, a CAAR will not be observed across all offspring if one or more offspring have insufficient informative SNPs on one, or both, sides of the recombination event (as in offspring 3 of Figure 2F), if such informative SNPs are beyond a pre-defined length threshold, or if an offspring has a true recombination event within the same interval (as in offspring 4 in Figure 2F). The issue of insufficient informative SNPs on one or both sides of a CAAR will be more prevalent in two cases. First, in offspring whose contribution from their other parent contains haplotypes that are in common with the haplotypes that the Parent possesses that are not in common with the CGP. This circumstance will result in large stretches of heterozygous, non-informative SNPs in the offspring, thereby preventing the observation of CAAR. Second, in cases where the parent has long stretches of homozygosity due to inbreeding. These cases would be evident in the SNP data and may influence the interpretation of the test.

At least two offspring would be necessary to observe CAAR, hence the POR-2 test can only be performed with at least two offspring. Some CAAR could occur by chance coinciding recombination, particularly when large intervals are lacking GOR-SNPs. Hence, the addition of more offspring increases the chance that the CAAR are due to a false CGP and at the same time also reduces the number of CAAR due to real coinciding recombinations in the offspring. Thus, confidence in the interpretation of the POR-2 test is contingent upon the number of CAAR observed and the number of offspring used to perform the test. The balance between these two factors was explored in the demonstration of the POR-2 test.

### Parent-offspring order resolution test 1 validation demonstration

When `Honeycrisp' was the phased individual and ‘Keepsake’ was the unphased individual, there were no GOR-haplotypes of ‘Keepsake’ that were composed of recombinant GOR-homologs from ‘Honeycrisp’ (File S1). There were 1627 GOR-SNPs available for this test. Conversely, when ‘Keepsake’ was the phased individual and ‘Honeycrisp’ was the unphased individual, GOR-haplotypes from 11 chromosomes of ‘Honeycrisp’ were composed of GOR-homologs from ‘Keepsake’ (File S1). There were 1929 GOR-SNPs available for this test. The regions at which the GOR-haplotype of ‘Honeycrisp’ showed recombination between GOR-homologs of ‘Keepsake’ perfectly matched the recombination points previously reported [3]. Thus, the interpretations of the POR-1 tests correctly matched the expectation of ‘Keepsake’ as the parent of `Honeycrisp'.

### Parent-offspring order resolution test 2 validation demonstration

The maximum cM thresholds evaluated for observed CAAR did not change the overall interpretations of any of the POR-2 tests when being equal to or larger than 10 cM (Table S1). Lower values would sometimes have led to inconclusive results. For instance, sometimes no CAAR were observed at a threshold value of 2 cM and 5 cM when the CGP was deemed false. This was the case with ‘Court Pendu Plat’ as CGP of the offspring of ‘Brabant Bellefleur’. Hence, the number of CAAR were reported below with a maximum allowed interval of 50 cM.

#### ‘Keepsake’ & ‘Honeycrisp’

When ‘Keepsake’ was evaluated as the CGP of the ten offspring of ‘Honeycrisp’, the number of CAAR observed across every pairwise combination ranged from 0 to 3 (Table S1). This range dropped to 0 to 2 when the number of offspring used for the test was increased to three. With ‘Honeycrisp’ as the CGP, the number of CAAR ranged from 9 to 15. These ranges changed to 0 to 2 and 9 to 13, respectively, when three offspring were instead used. When CAAR were evaluated across all offspring, the same pattern was observed where more CAAR were observed with ‘Honeycrisp’ as the CGP (Table S2). There were two separate instances with a single CAAR across four offspring with ‘Keepsake’ as CGP and ‘Honeycrisp’ as a Parent, however no instances with CAAR were observed across five or more offspring. With ‘Honeycrisp’ as CGP, there were eight instances of CAAR across all eight offspring of ‘Keepsake’. One additional CAAR was present across seven offspring, with the eighth offspring lacking informative SNPs on one side of the CAAR. Three additional CAAR were observed on chromosomes 8, 10, and 16 where one or two offspring possessed recombinations that coincided over the CAAR interval, obscuring the observation of CAAR in these offspring. The high number of CAAR across all offspring when ‘Honeycrisp’ was used as the CGP correctly indicated that ‘Honeycrisp’ is an offspring of ‘Keepsake’. Application of the test in the opposite direction resulted in a low number, often 0, of CAAR across groups of offspring of various sizes, which validates the conclusion of the foregoing test that ‘Keepsake’ is a parent of ‘Honeycrisp’ (see File S2 for all recombination information for all PO relationships evaluated).

#### ‘Reinette Franche’ & ‘nonpareil’

When ‘Reinette Franche’ was evaluated as the CGP of five offspring of ‘Nonpareil’, the number of CAAR observed across every pairwise combination ranged from 0 to 5 (Table S1). This range dropped to 0 to 1 when the number of offspring used for the test was increased to three. There were no instances where CAAR were observed across more than three offspring (Table S2). With ‘Nonpareil’ as the CGP of the six offspring of ‘Reinette Franche’, the number of CAAR across every pairwise combination of six offspring of ‘Nonpareil’ ranged from 3 to 11 (Table S2). This range dropped to 2 to 7 when three offspring were used instead. Two CAAR were observed across all offspring of ‘Reinette Franche’. An additional three CAAR were observed across four offspring, with the fifth offspring lacking informative SNPs on one side of the CAAR. Both directions of the test suggest that ‘Reinette Franche’ is a parent of ‘Nonpareil’.

#### ‘Brabant Bellefleur’ & ‘Court Pendu Plat’

With ‘Brabant Bellefleur’ as CGP of the five offspring of ‘Court Pendu Plat’, the number of CAAR across every pairwise combination of offspring ranged from 1 to 2 (Table S1). This range was 0 to 1 using every combination of three offspring of ‘Court Pendu Plat’, with no CAAR being observed across four or five offspring (Table S2). With ‘Court Pendu Plat’ as CGP of the five offspring of ‘Brabant Bellefleur’, the number of CAAR across every pairwise combination of offspring ranged from 5 to 10 (Table S1). This range dropped to 3 to 9 when the number of offspring was increased to three. Three CAAR were observed across all five offspring of ‘Brabant Bellefleur’ (Table S2). In addition, one additional CAAR was observed across four offspring where the fifth offspring lacked informative SNPs on one side of the CAAR, and three additional CAAR were observed across four offspring, where the fifth offspring possibly had a coinciding recombination event over the CAAR interval. Both directions of the test suggest that ‘Court Pendu Plat’ is an offspring of `Brabant Bellefleur'.

### Interpretation of the parent-offspring order resolution test 2

In a one-way application of the POR-2 test, interpretation of the number of CAAR observed would require the determination of generally applicable thresholds that allow for either an unambiguous determination on the direction of a PO duo or on the inconclusiveness of the test results. These thresholds would vary with the number of offspring used. The remainder of this section describes the establishment of a rubric and thresholds based on the observations from this study.

When at least four offspring were used for the POR-2 test, there were no instances of more than one CAAR observed with a true CGP, whereas there was a minimum of two CAAR observed across all offspring with a false CGP (Table 1, Table S2). Thus, in this study, the presence and absence of multiple CAAR were defining characteristics for rejecting or confirming a CGP, respectively, when at least four offspring were used to conduct the test. To be conservative, we recommend a minimum threshold value of three CAAR observed across four offspring for determining a CGP hypothesis to be false.

**Table 1:** Interpretation of POR-2 test results in apple based on the number of observed common areas of apparent recombination (CAAR)

**Number of offspring**	**Observed CAAR in the current study**		**CAAR thresholds for POR-2 test interpretation**		
	True CGP	False CGP	False	True	Inconclusive
	#Max	#Min			
≥4	1	2	≥3	0	1–2
3	2	2	≥4	0	1–3
2	5	3	≥6	0	at least 1–5

With the use of three offspring, a maximum of two CAAR was observed with a true CGP and a minimum of two CAAR was observed with a false CGP (Table S1). Thus, to allow for a high level of confidence in the test when using only three offspring, we recommend the threshold value for the maximum number of observed CAAR for confirming a true CGP be zero. There was only one instance where two CAAR were observed when the CGP hypothesis was true across all 140 combinations of three offspring evaluated in this study. Thus, to be conservative, we recommend a minimum threshold value of four CAAR observed across three offspring for determining a CGP hypothesis to be false.

With three or more offspring used to conduct the POR-2 test, additional analyses could be performed in cases of inconclusive test results. Instances could also be considered where one offspring in the grouping lacks informative SNPs on one side of a CAAR. With this extra step, the minimum number of CAAR observed with three offspring and a false CGP may have increased from three to four (Table S2), which matched our suggested threshold value for determining a CGP to be false when only using three offspring to conduct the test.

There was considerable ambiguity in the results when only two offspring were used for the test (Table S1), which made the establishment of thresholds for test interpretation difficult. For example, with ‘Reinette Franche’ as the CGP of the offspring of ‘Nonpareil’, a relationship deemed true, a maximum of five CAAR was observed, while when ‘Nonpareil’ was evaluated as the CGP of the offspring of ‘Reinette Franche’, a relationship deemed false, a minimum of three CAAR was observed (Table S1). These results are in opposition to the general trend and suggest that using only two offspring for the POR-2 test would be ill-advised unless stringent thresholds regarding the minimum and maximum number of CAAR observed were imposed for determining the CGP as true or false. Thus, to allow for a high level of confidence in the test when using only two offspring, we recommend the threshold value for the maximum number of observed CAAR for confirming a true CGP be zero and the observation of at least six CAAR to confirm a false CGP (Table 1, Table S2). However, because of such a high number of CAAR were not observed in many cases even with false CGPs, the POR-2 test will likely be too ambiguous to be effective with only two offspring.

## Discussion

### Interpretation of the parent-offspring order resolution test 1

The POR-1 test result was unambiguous (File S1) and thus should be the preferred option for ordering PO duo relationships. However, such an unambiguous result was only possible with accurately phased SNP data, which can only be achieved with many confirmed offspring and/or the availability of confirmed parents, which are not always available. The example included in this study to demonstrate the application of the POR-1 test, the PO duo relationship between ‘Honeycrisp’ and parent ‘Keepsake’, was included because accurate reference phasing data was available through a previous study [3]. This previous phasing data was considered sufficiently accurate for the POR-1 test because SNP data for both parents of both ‘Honeycrisp’ and ‘Keepsake’ and numerous offspring of both were used for the phasing. While this worked for the demonstration in this study, neither parent would be available in a real scenario involving an unordered PO duo. If a parent was known, a simple trio exclusion test could be used to order the relationship (ex. as was used in some cases in Muranty et al. [7]). Additionally, sufficiently accurate phasing may not be available for some individuals in unordered PO duo relationships due to a lack of available offspring, which would prevent a clear interpretation of the POR-1 test. A level of phasing considered sufficient for the POR-1 test was not established in this study. Instead, the “POR-2” was developed in response to this limitation.

### Interpretation of the parent-offspring order resolution test 2

The POR-2 test evaluates the number of CAAR across all chromosome pairs. It gave clear results across each PO duo when tested for both alternate genealogies with numerous offspring. However, the ability to test in both directions would not be possible in many other cases, which would result in some ambiguity in the interpretation of the test results. Hence, we developed a rubric for the further interpretation of the test results in one-way applications. To allow for a high level of confidence in the test, stringent thresholds regarding the minimum and maximum number of CAAR observed were imposed for determining the CGP as true or false (Table 1). The power of the test is determined by the magnitude of the difference between the number of observed CAARs with a true CGP due to a chance coincidence of true recombinations and the minimum number of observed CAARs with a false CGP due to artificial recombinations. The former rapidly declines with the increase of the number of offspring used in the test. Regrettably, the latter might also decline to some degree due to an increase in the likelihood of having an offspring with interrelated parents, which could result in some CAAR being unnoticeable. Coancestry between parents could result in an offspring with chromosomal regions that are identical to the Parent used in the test. If such a region in an offspring would include both a CAAR (identified across other offspring) and the end of a chromosome, then the CAAR would not be observed in the offspring. This was the case on chromosomes one and ten for offspring of ‘Reinette Franche’ (Table S2). This observation in offspring of ‘Reinette Franche’ could be due to ‘Reinette Franche’ being a particularly prolific ancestor of historical cultivars [7]. This was a likely factor why the minimum number of CAAR observed with a false CGP was observed between ‘Reinette Franche’ and ‘Nonpareil’, which in turn may serve as an approximate limit of three for the minimum number of observed CAAR to deem a CGP false when using only two offspring.

If POR-2 test results would be inconclusive, additional analyses could be performed to increase support for conclusions on the direction of a PO duo. First, CAAR present only across some of the offspring but not all could be considered where some of the total offspring have insufficient informative SNPs on one side of a CAAR, such as was previously described for offspring of ‘Reinette Franche’. From the results of this study across cases with four offspring, the minimum value of CAAR with a false CGP hypothesis increased from two to four if such cases were considered where one offspring lacked informative SNPs over an interval where a CAAR was observed in the other offspring. Additionally, the nature of observed CAAR could also be clarified if SNP data for the other parents of the offspring were used in the test.

In this study, the power of the POR-2 test was evaluated without additional data from other parents of offspring. However, in future application of this test, such additional data could be used to clarify which alleles in heterozygous SNPs of offspring came from the Parent used in the test, thus making some previously uninformative heterozygous SNPs informative. This could reveal some CAAR that would otherwise not have been observed and to clarify intervals of recombination. In the present study, this information would have revealed that some offspring of ‘Keepsake’ had concurrent recombinations close to, or occurring over CAAR intervals on chromosomes 8, 10, and 16 (data not shown) and possibly also could have occurred in some offspring of ‘Reinette Franche’ on chromosomes 7 and 16 and in some offspring of `Brabant Bellefleur' on chromosomes 3 and 9 (Table S2).

Factors other than the number of CAAR were also considered for the interpretation of the POR-2 test but were ultimately rejected. CAAR interval length was considered as an attribute that could help confirm or deny a POR-2 test hypothesis, as the presence of CAAR over very short intervals would be highly unlikely to represent chance recombinations. However, the need for using GOR-SNPs resulted in most CAAR occurring over longer distances (see Table S3). Additionally, while the presence of CAAR over short intervals may be indicative of a false CGP, it could be too that these represent recombination hot spots in breeding germplasm due to human selection efforts to combine favorable alleles of co-localizing QTL for traits of economic interest, and thus could lead to false conclusions. In apple, such a QTL hotspot occurs at the proximal end of chromosome 16, which harbors major QTL for fruit quality traits like acidity (*Ma*) [14], texture [15], bitter pit [16, 17] (Bp-2), and cracking [18]. In Cherry, which is a species that could conceivably use the POR-2 test, such a QTL and recombination hot-spot was reported on chromosome 2 [19]. Formal significance testing for the POR-2 test was also considered but not utilized because of the uncontrollable variable levels of SNP informativeness that exist across individuals. The variable level of SNP informativeness would mostly be due to complex interrelations between individuals evaluated in these tests that can lead to stretches without informative SNPs and prevent the observation of CAAR.

### Caveats to parent-offspring order resolution tests

Despite the successful interpretations of the POR tests in the demonstrations provided, the specifics of the results regarding the number of CAAR and offspring for POR-2 test interpretation would only be appropriate for similar datasets in apple. Though these specifics should show similar trends in other species, differences in SNP densities, differences in the quality and coverage of the genetic map used, and the peculiarities specific to different species could impact the interpretation of the POR-2 test. *Vitis vinifera*, for example, has 19 chromosomes [20], which would provide two additional chromosomes to use for the POR-2 test, possibly leading to a slight increase in the differentiation in average CAAR observed between false and true CGPs. Extreme selfing and/or extreme inbreeding, which were not encountered in the present study, would have a profound impact on the implementation and interpretation of the POR tests. These impacts could be explored in a species that has these attributes, such as peach (*Prunus persica*). Finally, these POR tests were only possible with very highly curated and accurate allele calls and a dense and accurate genetic map covering most of the length of the genome. If these prerequisites are not met, the tests either cannot be performed or would have less trustworthy results. The tests should be possible with sufficiently dense re-sequencing data, though a curation step analogous to the SNP array data curation steps used in the present study, as errors in sequence and/or alignment or rare mutations could negatively influence the test interpretation. Sequence data that is low coverage (for example, typical genotype-by-sequencing data) would not be suitable for the POR tests because this type of data would have numerous instances where an allele call would be falsely homozygous due to insufficient local coverage depth. These false allele calls would violate the prerequisite of curated and accurate allele calls.

### Relevance of newly ordered PO relationships

The POR-2 tests determined that ‘Reinette Franche’ was a parent of ‘Nonpareil’ and ‘Brabant Bellefleur’ was a parent of ‘Court Pendu Plat’. These specific cases were chosen for testing from Muranty et al. [7] because there were at least five offspring available for each and because all four cultivars were ancient with sometimes imprecise or ambiguous provenance information. ‘Reinette Franche’ and ‘Nonpareil’ were recorded as originating in 1540 [21] and mid-1500s [22], respectively. If the record for ‘Reinette Franche’ is correct, the results of the POR-2 test mean that ‘Nonpareil’ cannot be older than the mid-1500s and that its recorded origin time may be correct. Resolution of this pedigree will be useful for inheritance studies, as ‘Reinette Franche’ was identified as a particularly common pedigree ancestor of historic and some modern cultivars [7].

‘Court Pendu Plat’ was first described in 1613, though it is thought to be much older and ‘Brabant Bellefleur’ is described as originating in the late 1700s^22^. This recorded provenance was why Muranty et al. [7] listed ‘Court Pendu Plat’ as the parent of ‘Brabant Bellefleur’. The results of the POR-2 test strongly suggest that the opposite relationship is true, which greatly pushes back the date of origin of ‘Brabant Bellefleur’. This result also highlights the need for such a test to clarify imprecise provenance information.

### Conclusion

The successful demonstrations of the POR tests suggest they will be of great use to future pedigree reconstruction studies. These tests are currently successfully being used in an ongoing large-scale apple pedigree reconstruction project [13]. The POR tests could be adapted to other species, however caveats like extreme inbreeding, selfing, and concerns regarding SNP call or marker order and density may need to be considered.

## Methods

### Plant material and genotypic data

A set of 50 accessions was used in this study (the list, relevant metadata, and curated SNP data are in Table S3). All individuals were genotyped on either the Illumina Infinium apple 20 K SNP array [23] or the Affymetrix 480 K SNP array [10] (listed in Table S3). Processing of the SNP data and the integration of the data from both SNP arrays were conducted as described in Howard et al. [24]. SNP calls for between 8348 and 10 293 SNP markers were available for each individual. The genetic map used was an augmented version of the 20 K iGL map [10], described in Howard et al. [24].

### Parent-offspring order resolution test 1 application

SNP phasing for the POR-1 test was performed using FlexQTL software (www.flexqtl.nl) [25]. The phasing capabilities of FlexQTL have been demonstrated in numerous studies, (e.g. [3, 4, 25, 26],). The POR-1 test was demonstrated on the known relationship of ‘Keepsake’ as a parent of ‘Honeycrisp’. Both ‘Honeycrisp’ and ‘Keepsake’ were separately phased using their known offspring listed in Table S1. The parents of ‘Honeycrisp’ and ‘Keepsake’ were not used for phasing of either cultivar because if this test were conducted using a pair of individuals of unknown pedigree order, no parents of either individual would be available for phasing, and we wanted to ensure our demonstration test results would be consistent with this reality. However, the parents of all offspring were included for improved phasing of both ‘Honeycrisp’ and ‘Keepsake’. The POR-1 test was conducted using an automated Excel tool (File S1) suitable for the current dataset. The test was conducted in both directions, with one individual serving as the unphased individual and the other as the phased individual in one test and the roles reversed for testing in the opposite direction.

### Parent-offspring order resolution test 2 application

The POR-2 test was designed for a one-way application. However, here the validity of its approach was demonstrated in a two-way application; that is, by the testing both alternative genealogies of each PO relationship. The two-way testing was possible for each PO duo because at least five offspring were available for each individual in each duo. Conclusions on the true direction came from the relative number of CAAR observed for both genealogies within a PO duo.

As an initial demonstration, the POR-2 test was applied to the PO duo relationship of known order, ‘Keepsake’ as a parent of ‘Honeycrisp. The test was then applied to the PO duo relationship of unknown order between ‘Reinette Franche’ and ‘Nonpareil’, and the PO duo relationship of assumed order of ‘Court Pendu Plat’ as a parent of ‘Brabant Bellefleur’, which was based solely on provenance information [7]. For all three PO duos, the POR-2 test was conducted with one individual as CGP, and again with the other individual as CGP. The number of available offspring were eight for ‘Keepsake’, 10 for ‘Honeycrisp’, six for ‘Reinette Franche’, and five for ‘Nonpareil’, ‘Court Pendu Plat’ and ‘Brabant Bellefleur’. To probe the limits of the POR-2 test, every pairwise comparison of two, three, and then all offspring were made for each PO duo. The minimum of two offspring was chosen because the inclusion of at least two offspring in the test would be necessary to observe CAAR. CAAR were recorded for pairs, triplets, and across all offspring and all chromosomes over cM threshold lengths of less than 2 cM, 5 cM, 10 cM, 15 cM, 20 cM, and 50 cM. An *ad hoc* Microsoft Excel tool was used to perform the POR-2 test (File S3). Output from this tool was processed to identify CAAR for test interpretation. CAAR intervals for pairs and triplets of offspring (File S2) were considered to be the maximum distance over recombination intervals that included a CAAR. For example, if one offspring had a recombination interval between 28.14 cM and 32.54 cM and a second offspring had a recombination interval between 30.12 cM and 52.19 cM, the CAAR interval would be 52.19 cM - 28.14 cM = 24.05 cM. This distance was used for reporting rather than the distance over which the CAAR would have been needed to be present if a CGP was indeed false (32.54 cm - 30.12 cM = 2.42 cM in this case) to be more conservative and consider the more likely possibility that chance coinciding recombinations had occurred over such long distances. However, CAAR intervals over all offspring (as in Table S2) were reported as the distance over which recombination would have taken place across all offspring for which a coinciding recombination occurred if the CGP was false. This was used instead because as more offspring were included in the test, false CAAR across all offspring were increasingly rare yet the chance that one or more offspring would have extensive stretches of uninformative SNPs on one or both sides of the CAAR would also increase, thus increasing a CAAR’s apparent length even if the CGP were false.

## Acknowledgements

Funding for this research was provided by the Niedersächsisches Ministerium für Wissenschaft und Kultur through the EGON project: “Research for a sustainable agricultural production: Development of organically bred fruit cultivars in creative commons initiatives” (nr. 3250), the USDA NIFA Specialty Crop Research Initiative projects, “RosBREED: Enabling marker-assisted breeding in Rosaceae” (2009-51181-05808) and “RosBREED 2: Combining disease resistance with horticultural quality in new rosaceous cultivars” (2014-51181-22378), and the USDA NIFA Hatch program through the Agricultural Experiment Station - University of Minnesota Project MIN-21-040. The authors would like to thank Cameron Peace for reviewing an early version of the manuscript, the authors of Muranty et al. [7] for sharing some data prior to their publication for use in this study, the Fondazione Edmund Mach for sharing some 20 K SNP array data used in the study, and the organizations Seed Savers Exchange in Decorah, Iowa, USA, and Ökowerk in Emden, Germany for providing germplasm used in the study.

## Author Contributions

N.P.H., J.J.L, and E.v.d.W conceived and designed the study. N.P.H performed SNP data curation and analyses. N.P.H. and E.v.d.W wrote the manuscript. All authors critically reviewed the manuscript and approved of the final version of the manuscript.

## Data availability

All data supporting the results of this study are present in the supplementary files.

## Conflict of interest statement

The authors declare no competing financial interests.

## Supplementary data

Supplementary data is available at *Horticulture Research Journal* online.

## Supplementary Material

Web_Material_uhab069
